# Foot-Sweats

**Published:** 1885-04

**Authors:** E. Fornias

**Affiliations:** 401 Pine Street, Philadelphia


SILICA.—*Offensive foot-sweat* with rawness between the toes. Itching of soles, driving to despair.

SEPIA.—*Profuse foot-sweat* or very *fetid*, causing soreness of toes. Burning, or heat of the feet at night. Crippled nails.

BARYTA CARB.—*Fetid foot-sweat*, with callosities on the soles which are painful on walking. Soles feel bruised at night, keeping one awake, after rising and walking.

LYCOPOD.—*Profuse and fetid foot-sweat*, with burning in the soles. One foot hot, the other cold, or both cold and sweaty. Swelling of the soles; they pain when walking. Fissures on the heels.

THUJA.—Fetid sweat on toes, with redness and swelling of the tips. Nets of veins, as if marbled, on the soles of the feet. Suppressed foot-sweat. Nails crippled, brittle, or soft.

GRAPHITES.—Profuse foot-sweat, not fetid as in Sep. or Silica, but the most moderate walking causes soreness between the toes, so that the parts become raw. Spreading blisters on the toes, thick and crippled toe nails. (Jahr gives fetid feet under Graph.)

KALI CARB.—*Profuse fetid foot-sweat.* Swelling and redness of the soles; chilblains. Stitches in the painful and sensitive corns.

CARBO VEG.—*Foot-sweat* excoriating toes. Toes red, swollen. Stinging, as if frosted. Tip of toes ulcerated.

ZINCUM.—The feet are sweaty and sore about toes; also fetid. Chilblains from scratching and friction. The suppression of sweat causes paralysis of the feet.

MUR. ACID.—Cold sweat on the feet, evening in bed. Swelling, redness, and burning of tips of toes. Chilblains.

NITRIC ACID.—Foul smelling *foot-sweat.* Chilblains on the toes.

CALC. OST.—*Foot-sweat* which makes the feet sore. Feet feel cold and damp, as if she had wet stockings. Burning in the soles.

LACTIC ACID.—Profuse *foot-sweat*, but not fetid (Graph.).

SULPHUR.—Sweating and coldness of the soles. Burning soles, wants them uncovered.

PETROL.—Feet tender and bathed in a *foul moisture.* Feet swollen and cold. Hot swelling of the soles, with burning. Heel painfully swollen and red. Chilblain. Tendency of skin to fester and ulcerate.

IODUM.—Acrid, corrosive *foot-sweat.* Œdematous swelling of the feet.
PLUMBUM.—*Fetid foot-sweat.* Swelling of the feet.
PODOPHY.—Foot-sweat evenings.
CANTHAR.—Temporary cold sweat on feet. Smells like urine.
HELLEBOR.—Humid, painless vesicles between the toes.
SQUILLA.—*Cold foot-sweat.* Sweat only on toes. Soles red and sore when walking. (Jahr gives the following.)
FOOT-SWEAT.—Acon. *Amm.* Baryt.° CALC. *Carb. veg.* Cocc. *Cup.* Cycl. *Dros.* Graph. Iod.° KALI. Kreos. *Lach.*° LYCOP. *Mag. m.* Merc. *Nat. m. Nit. ac. Nux jug. Petr.* Phos. *Phos. ac.* Plumb. *Puls. Sabad. Sabin.*° SEPIA.° SILIC.° SQUILL. Staph. °SULPH. *Thuj. Zinc.*
—CORROSIVE.—Iod. Lycop. Nit. ac. Silic. Zinc. (Carbo veg.).
—FETID.—Amm. *Baryt. Cycl. Graph. Kali.*° *Nit-ac.*° *Nux-jugl.* Phosph. Plumb. *Sep.* SILICA. *Zinc.*
—COLD.—Cocc. Dros. Ipecac. *Lycop.* Merc. *Squill.* Staph. *Sulph.* (Canth.).
—NIGHT (at).—Coloc. (Evening, Mur ac. Podoph.).
—SUPPRESSED.—°*Cup.* °*Kali.* °*Nat-m.* °*Nit-ac.* °*Sep.* °SILICA. (Apis. Rhus tox. Puls. Thuj. Zinc.).
—SOLES OF FEET (on).—Acon. Arn. Kali. Nat-m. *Nit-ac.* Petrol. *Plumb. Sabad.* Silica. Sulph.
—TOES (between the).—Acon. Arn. Clem. Cycl. Ferr. Kal. Sep. Sil. Squilla. Tarax. Thuja.

To this we may add :

—PROFUSE.—Carbo v. Graph. Kali c. Lactic ac. Lycop. *Sepia.*
—With much *itching* of soles.—Silica. Sulph.
—With *burning.*—Calc. Ost. Lycop. Mur. ac. Petrol. Sep. Sulph.
—With *rawness.*—Graph. Silica. (Carbo veg.).
—With *soreness.*—Baryta c. Calc. Carbo veg. Graph. Iod. Petrol. Sep. Squilla. Zinc.
—With *redness and swelling* in the soles.—Iod. Kali carb. Lycop. Petrol. Squilla—in the feet, Plumb.—in the tips, Mur. ac. Thuja—in the toes, Carbo veg.
—With *pain,* on walking.—Baryta c. Graph. Lycop. Squilla —at rest, Carbo veg. (stinging), Petrol. (pain in heel of foot.)
—With crippled nails.—Graph. Sepia. Thuja.
MOISTURE (rather than sweat).—Fetid, Petrol.—Cold, Calc.

E. FORNIAS, M. D.
401 PINE STREET, PHILADELPHIA.

